# Can Organic Solar Cells Beat the Near-Equilibrium Thermodynamic Limit?

**DOI:** 10.1021/acs.jpclett.2c01565

**Published:** 2022-07-13

**Authors:** Tanvi Upreti, Constantin Tormann, Martijn Kemerink

**Affiliations:** † Complex Materials and Devices, Department of Physics, Chemistry and Biology (IFM), Linköping University, 581 83 Linköping, Sweden; ‡ Centre for Advanced Materials, Heidelberg University, Im Neuenheimer Feld 225, 69120 Heidelberg, Germany

## Abstract

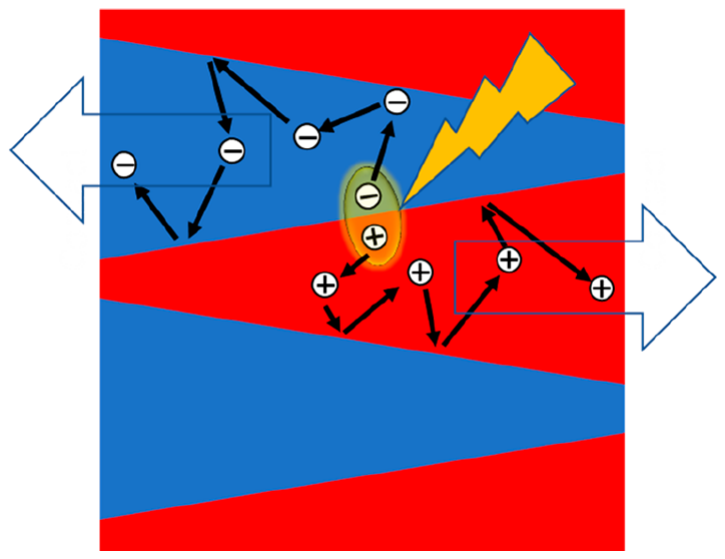

Despite an impressive increase over the past decade, experimentally determined power conversion efficiencies of organic photovoltaic cells still fall considerably below the theoretical upper bound for near-equilibrium solar cells. Even in otherwise optimized devices, a prominent yet incompletely understood loss channel is the thermalization of photogenerated charge carriers in the density of states that is broadened by energetic disorder. Here, we demonstrate by extensive numerical modeling how this loss channel can be mitigated in carefully designed morphologies. Specifically, we show how funnel-shaped donor- and acceptor-rich domains in the phase-separated morphology that are characteristic of organic bulk heterojunction solar cells can promote directed transport of positive and negative charge carriers toward the anode and cathode, respectively. We demonstrate that in optimized funnel morphologies this kinetic, nonequilibrium effect, which is boosted by the slow thermalization of photogenerated charges, allows one to surpass the near-equilibrium limit for the same material in the absence of gradients.

The past decade has witnessed a continuous rise in power conversion efficiencies (PCE) of organic solar cells, now reaching 19%.^1,2^ The bulk heterojunction (BHJ) approach, in which donor and acceptor compounds are intimately mixed in a single active layer, has enabled achieving this milestone by providing a means to split photogenerated excitons with near unity (internal) quantum efficiency into charge transfer (CT) states and subsequently into free charges.^3^ In many organic photovoltaic (OPV) systems, the required driving force, given by the difference of the (optical) bandgap *E*_*g*_^*opt*^ and the energy of the CT state *E*_*CT*_, leads to significant energy losses that show up as open circuit voltages (*V*_*OC*_) that are lower than those of inorganic solar cells of the same bandgap.^4^ It was predicted on the basis of numerical simulations^5^ and subsequently shown experimentally that careful tuning of energy levels allows free charge generation at minimal energy loss.^6^

Taking the Shockley–Queisser limit, which is derived for an ideal photovoltaic solar cell operating near thermodynamic equilibrium,^7^ as a reference point, two other factors can be identified that contribute to the generally low *V*_*OC*_ values of OPV systems. First, nonradiative recombination provides an additional loss channel on top of the unavoidable radiative recombination.^8,9^ Although the procedures that are used to experimentally determine the associated losses have been questioned,^10,11^ typical values for state-of-the-art OPV systems are estimated to be around 0.7 eV, and there is a considerable ongoing effort to reduce these losses.^4,9,12,13^ For the present work, which is about concept development, nonradiative losses are irrelevant in the sense that they, for a given material system, provide a more or less constant background loss. Here, and in the following, recombination refers solely to the annihilation of an electron–hole pair under the emission of photons or vibrations.

The second loss channel is specific to organic solar cells and is caused by the energetic disorder that broadens the relevant, low-energy parts of the electron and hole densities of (localized) states (DOS). Commonly employed treatments of this effect, which also leads to broadening of the absorption onset,^10^ assume near-equilibrium, that is, the electron and hole populations are assumed to be in thermal equilibrium with the lattice but not with each other.^14,15^ Although the on-site thermalization of photogenerated charges completes in picoseconds,^16,17^ the subsequent thermalization of the photogenerated electron and hole populations inside the DOS takes much longer.^18^ The physical reason is that sites that correspond to typical equilibrium energies sit deep in the DOS tails and therefore are relatively rare and take long to be found by the thermalizing charges. Since even in state-of-the-art OPV systems the energetic disorder amounts to several times the *k*_B_*T* value,^19^ the associated thermalization times are in the microsecond range and exceed the charge carrier lifetime in the device.^20^ In other words, the condition of near-equilibrium operation, on which also the Shockley–Queisser model is based, is not fulfilled in typical OPV systems, implying that they are not formally bound by the limits posed by near-equilibrium thermodynamics. Although this complicates the description of OPV devices, it is in principle good news. In a recent paper, we could show that the slow thermalization of photocreated charges leads to *V*_*OC*_ values that are 0.1–0.2 V higher than when thermalization would complete instantaneously.^19^ Nevertheless, thermalization is still a loss process that one would like to mitigate.

While the bulk heterojunction architecture provides a large interfacial area between the donor and acceptor moieties to facilitate charge separation, it does not generally provide a preferential direction of motion for the thermalizing charge carriers. Consequently, thermalization is mostly associated with undirected, diffusive motion.^21^ In a recent paper, it was shown that the introduction of a vertical (in the direction of current flow) gradient in the donor:acceptor ratio leads to a rectification of this diffusive motion, leading to improved charge separation and extraction and suppressed surface recombination in full devices.^22^ In absence of a built-in voltage, the broken inversion symmetry due to the composition gradient still leads to a finite open circuit voltage. The slow thermalization of photocreated charges leads to *T*_*eff*_ ≫ *T*, which significantly boosts the effect of the composition gradient.^22^ A short derivation of an expression for the resulting open circuit voltage is given in section 1 of the Supporting Information.

Composition gradients in OPV have been investigated in some detail, both in experiments^23−36^ and simulations.^23,37,38^ These studies aimed for improved charge transport and reduced leakage currents based on the idea that a compositional gradient effectively propels electrons and holes to their corresponding electrodes.^25^ That said, most experimental studies reported at least slightly enhanced performances, mainly due to increased *j*_*SC*_ and improved fill factors, for graded OPV as compared to nongraded ones.^24,31,32,39^ Chen et al. even observed increasing *V*_*OC*_ for increasing gradient strength, but, not considering the slow thermalization processes, they attributed the effect to the additional chemical potential energy gradient that adds to the built-in potential of the device.^25^ The topic of composition gradients gained additional urgency with the very recent advances in OPV performance made through sequential deposition of the donor and acceptor compounds.^32−36^ Apart from being robust, this processing technique is prone to lead to strong composition gradients, the effect of which so far lacks a formal interpretational framework. With the exception of the work by Andersson and Kemerink,^22^ simulations of graded OPVs only utilized drift-diffusion models, neglecting both slow relaxation processes and morphology aspects, and did not lead to a consistent physical picture.

Here, we numerically investigate the achievable performance increases by introducing composition gradients of various types in the bulk heterojunction morphology. In systems without phase separation, that is, molecularly mixed BHJs, we investigated several composition profiles and find, for stronger gradients, considerable differences in terms of the achievable open circuit voltages and short circuit current densities *j*_*SC*_. Although the gradient devices systematically outperform the corresponding homogeneous devices, their power conversion efficiencies (PCE) lie below the near-equilibrium limit of the corresponding material. In marked contrast, devices where the composition gradient is implemented in the form of a phase-separated funnel morphology, the PCE can exceed the corresponding near-equilibrium value. This is possible because the devices operate far from equilibrium.

We use a kinetic Monte Carlo model that has been described in detail elsewhere.^19,40^ In short, it implements the extended Gaussian disorder model on a cubic lattice for a full solar cell including ohmic contacts. Charge transport is described as a Miller–Abrahams hopping process. In addition, the model includes the dynamics of excitons (with binding energy 0.5 eV) and interfacial CT pairs. Effective recombination rates are used to account for both radiative and nonradiative recombination, as discussed in Supporting Information, section 3.

Full Coulomb interactions, including those by image charges in the electrodes and those resulting from the periodic boundary conditions in the lateral directions are considered. In simulations, we used realistic and symmetric hopping parameters for both electrons and holes, viz. a Gaussian disorder σ = 75 meV, an attempt-to-hop frequency ν_0_= 3 × 10^11^ s^–1^ and a nearest neighbor distance of *a*_*NN*_ = 1.8 nm that also determines the CT binding energy as *E*_*b*,*CT*_ = *q*/4*πε*_0_ε_*r*_*a*_*NN*_≈ 0.22 eV.^41^ Hopping to non-nearest-neighbors was allowed up to a cutoff radius of . The highest occupied molecular orbital (HOMO) and lowest unoccupied molecular orbital (LUMO) energy levels of the donor (acceptor) were taken 5.3 eV/3.3 eV (5.7 eV/3.7 eV), giving the system an effective band gap of 1.6 eV. The anode and cathode work function were set at 5.0 and 4.0 eV to give a built-in voltage *V*_*bi*_ = 1.0 V; for symmetric devices, both work functions were set at 4.5 eV (*V*_*bi*_ = 0 V). A lattice temperature of 300 K and a uniform generation rate corresponding to roughly 1 Sun (*G* = 10^28^ m^–3^ s^–1^) were used. The simulation box size was 30 × 30 × 50 sites,^3^ giving an active layer thickness of 90 nm for all calculations. Since the focus of this study was to investigate the impact of composition gradients, other factors impacting *V*_*OC*_ have not been addressed. A full list of parameters entering the kMC model is given in Supporting Information, section 3, where also the consistency of the model and its parameters with detailed balance is discussed.

Before turning to phase-separated gradient morphologies, we investigated different (laterally) homogeneous composition profiles. That is, apart from the composition gradient in the current (*z*-) direction, no further inhomogeneity is present. The full results can be found in Supporting Information, section 2. In absence of phase separation, the gradient strength is denoted as  where  is the donor concentration at the anode. Hence, a 90:10 gradient runs from 90% donor material at the anode to 10% at the cathode, and vice versa for the acceptor material. The results show that significant increases in especially *V*_*OC*_ can be realized with suitably chosen nonlinear composition profiles. In the following, we therefore consider the optimal homogeneous profile, which has the shape of the Fermi-distribution, to compare to the phase-separated morphologies. Specifically, integrating the optimal composition gradient eq S10 in a kMC simulation of a full device, i.e., with finite built-in voltage, leads to the red *j–V* curve in Figure 1a. In line with the findings of Andersson, the composition gradient leads to an enhancement in device performance as compared to the same device without composition gradient (black).^22^ At the same time, the absence of phase separation leads to a large interfacial area and concomitantly to a strong CT recombination, see the open red inverted triangles in Figure 1b. This explains the poor fill factors (0.43 and 0.41) and modest overall power conversion efficiencies (4.1% and 5.8%) in these devices (Supporting Information, Table S2).

**Figure 1:**
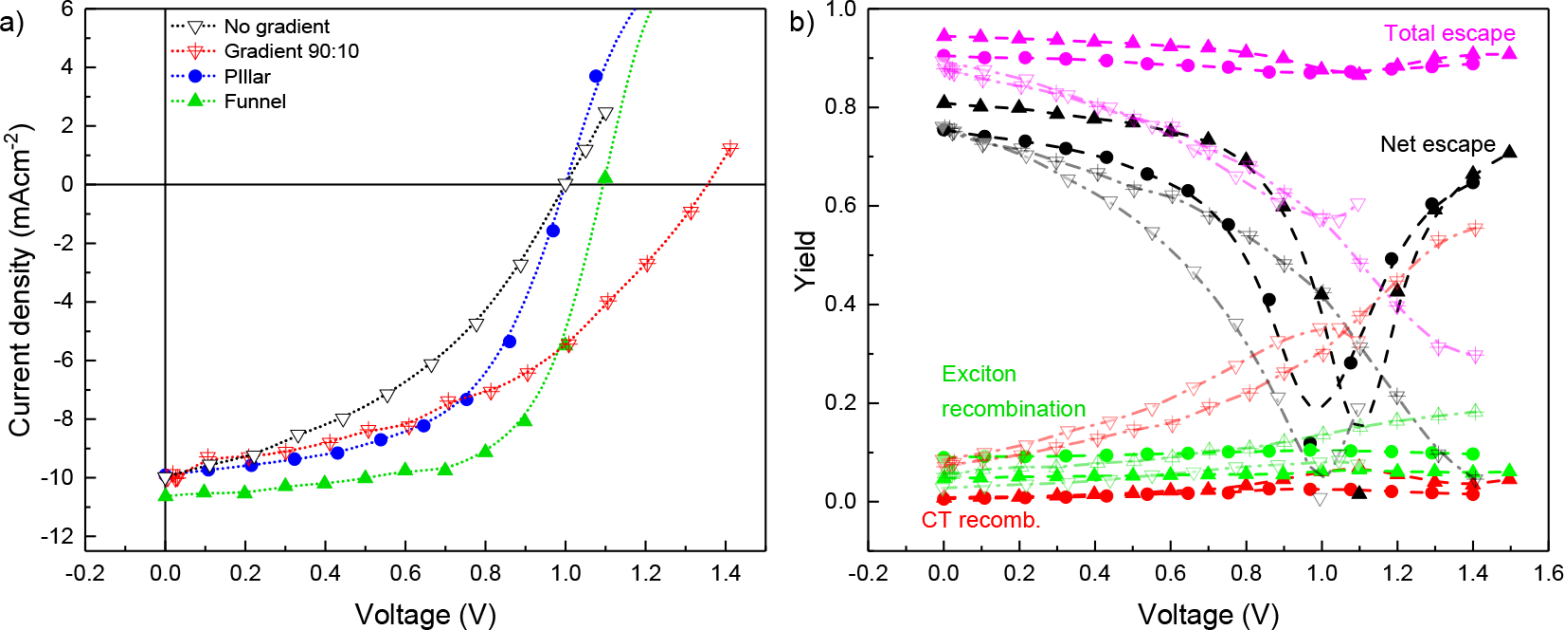
a) *j*–*V* curves calculated by kMC for four different active layers: no phase separation, no gradient (open inverted triangles); no phase separation, 90:10 gradient (open inverted crossed triangles); 10–7 pillar morphology (circles) and 10–3 funnel morphology (closed triangles, cf. Figure 2). *V*_*bi*_ = 1 V. (b) Corresponding yields for the different morphologies in panel a. Total and net escape yields are defined as *y*_total_ = (*J_n,_*_an_ + *J_n,_*_cat_ + *J_p,_*_an_ + *J_p,_*_cat_)/*J*_abs_ and *y*_net_ = (−*J_n,_*_an_ + *J_n,_*_cat_ + *J_p,_*_an_ – *J_p,_*_cat_)/*J*_abs_, where *J*_(*n/p*),(an*/*cat)_ is the current density of photogenerated electrons/holes extracted via the anode/cathode and *J*_abs_ is the current density corresponding to light absorption. The curves labeled exciton and CT recombination show the relative current densities associated with exciton and CT recombination, i.e., the fraction of photogenerated charges that undergo these processes.

In ref (40), we have shown that the complicated phase-separated morphology of actual OPV devices can be mapped on a simplified pillar-type morphology with a similar characteristic length scale, in the sense that the *j*–*V* curves and the recombination transients of the device can then be adequately reproduced from the resulting kMC model. Indeed, also for the parameters used here, the device labeled as a 10–7 pillar in Figure 1a shows a much-improved performance compared to the device without phase separation and with zero gradient. Here, 10–7 denotes a 10 × 10 × 50 unit cell of donor material with 7 × 7 × 50 inclusions of acceptor material. Hence, the 30 × 30 device area is covered by 3 × 3 unit cells that stand parallel to the (*z*) current direction. Note that the 10–7 pillar morphology corresponds almost exactly to a 50:50 D:A composition since (10^2^-7^2^):7^2^ = 51:49.

To combine the advantages of a phase-separated morphology with those of a gradient composition, we implemented a tapering of the pillars from top to bottom, giving rise to a funnel-shaped morphology as illustrated in Figure 2 (middle panel). Here, 10–3 refers to a morphology where the top surface of a 10 × 10 unit cell of donor material has a 3 × 3 inclusion of acceptor, turning to a 10 × 10 unit cell of acceptor material with a 3 × 3 inclusion of donor material at the bottom surface. By rotating the inclusions in the *xy*-plane by 45°, this leads to a linear composition gradient that, for 10–3, roughly corresponds to 90:10 (since (10^2^–3^2^):3^2^ = 91:9). A three-dimensional visualization of this morphology can be found in Supporting Information, section 4. Clearly, the resulting (simulated) device (PCE 7.3%) outperforms both the pillar morphology (PCE 6.2%) as well as the gradient device without phase separation. A detailed analysis of the yields of the various processes in Figure 1b shows that the improvement over the pillar device is mostly due to a much-suppressed diffusion loss, i.e., charge carriers ending up at the wrong contact and recombining. In Figure 1b, this loss channel shows up as the difference between the total (magenta) and net (black) escape yields, where the former (latter) reflect the fraction of photogenerated charges that reaches any of the contacts (the desired contact). This finding is consistent with the funnel morphology channeling highly diffusive “hot” charges toward the desired electrode.

**Figure 2:**
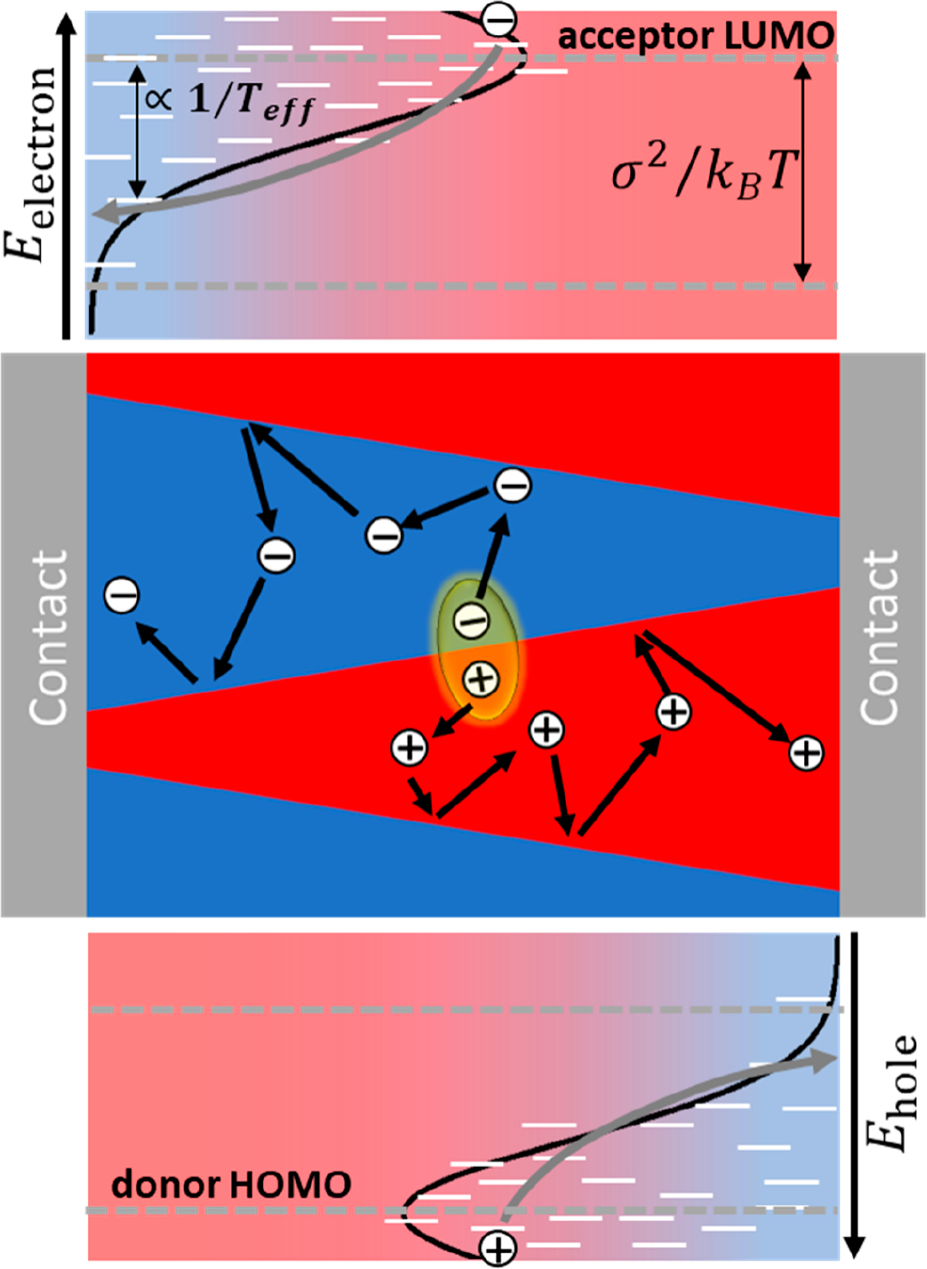
Schematic illustration of the directed diffusion of hot photogenerated electrons (upper panel) and holes (lower panel) by a phase-separated funnel morphology (middle panel). The increasing site density of donor (red) and acceptor (blue) phases leads to a directed stochastic motion of holes (electrons) to the right (left). The Gaussian distributed hopping sites are indicated by white lines, the incomplete thermalization of charges by gray arrows and the light red-to-blue shaded colors.

In ref (19), we have shown that the kMC input parameters can be used to calculate near-equilibrium values for *V*_*OC*_ and fill factor, following the framework laid out by Rau.^42^ Here, we will extend this methodology to calculate the near-equilibrium upper limit for performance for a homogeneous device with the same absorption spectrum and short-circuit current, but with instantaneous and complete thermalization of the charge carrier populations. In Supporting Information, section 3, we show that, in the limit of vanishing disorder, the outcomes of kMC model are consistent with those of the near-equilibrium model.

We start from the extended Shockley equation^43^
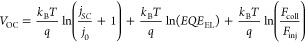
1

Here, the first term on the right-hand side is the radiative limit for *V*_OC_. The second term accounts for losses due to nonradiative recombination that are absent in the idealized materials typically considered in Shockley–Queisser-type models and will be ignored from here on; including the EQE value assumed in our (idealized) kMC simulations would lead to a reduction of the predicted near-equilibrium *V*_OC_ by ∼0.08 V. The third term was derived by Kirchartz et al. and accounts for differences in collection and injection efficiency.^43^ Using explicit calculations as described in ref.^19^ we typically find *F*_coll_ ≈ *F*_inj_ for the devices studied here, so only the first term of eq 1 remains.

The reverse dark saturation current can be obtained as the overlap of the external photovoltaic quantum efficiency spectrum with the blackbody spectrum  as
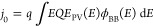
2

Here, *EQE*_PV_ is related to the internal photovoltaic quantum efficiency through *EQE*_PV_(*E*)=*IQE*_PV_(*E*)ϕ_abs_(*E*). Since we are interested in an upper limit, we set *IQE*_PV_ = 1, which is also a reasonable approximation for OPV.^19^ Due to the steepness of the blackbody spectrum, only the energetically lowest parts of the CT and S1 contributions to the absorption spectrum ϕ_abs_ are important. We can therefore write ϕ_abs_ as
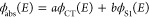
3where the CT and S1 singlet absorption spectra are calculated as convolutions of the relevant HOMO and LUMO levels.^10^ Their central energies are corrected for the Coulomb binding energies of the S1 and CT states. Since typical organic semiconductors are strong absorbers, we take *b* = 1 at the absorption maximum. The factor *a* can then be estimated from
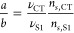
4where ν_S1_ = 1 × 10^9^ s^–1^ and ν_CT_ = 3 × 10^7^ s^–1^ are the total S1 and CT recombination rates as used in the kMC model, cf. Table S1. Equation 4 makes the reasonable assumption that CT and S1 recombination are competing against the same or at least similar loss channels, such that their relative lifetimes reflect their relative oscillator strengths. The second term on the right-hand side of eq 4 accounts for the fact that the number of absorption sites in the simulation box, *n*_*s*_, is different for S_1_ and CT absorption, which is calculated for each morphology considered in this paper.

Applying this methodology to the 10–3 funnel device leads to a near-equilibrium *V*_*OC*_ value of 0.92 V, which is indeed below the kMC value of 1.09 V; the 0.17 V difference highlights the importance of nonequilibrium effects. Note that a similar analysis for a symmetric 10–7 pillar morphology leads to *V*_*OC*_ = 0.92 V versus 1.00 V for the value from the *j*–*V* curve. As expected, the 0.08 V difference is less than for the asymmetric funnel device, but not zero due to the incomplete thermalization of photogenerated charges.^19^ For the 10–3 funnel device, the PCE is 7.3%, which is below the near-equilibrium limit of 8.5% for the same absorption spectrum and *j*_*SC*_.

Next, we performed a systematic variation of the funnel geometry in varying the gradient strength with constant unit cell size (in two series 10–*n* and 15–*n* with *n* = 0–3) and in varying the unit cell size with constant apex size (in a series *m*–1 with *m* = 6, 8, 10, 12, and 15). For the former series, we find a crossover between *n* = 1 and *n* = 2, where for smaller *n* (*n* = 0 and *n* = 1), the device power conversion efficiency exceeds the near-equilibrium limit of the same material in absence of gradients and under near-equilibrium conditions. Hence, we evaluate the near-equilibrium limit for a material with the same disorder-broadened absorption profile as used in the kMC simulations and not the rectangular profile assumed by Shockley and Queisser. This phenomenon persists for all *m* in the latter series where *n* = 1, as shown in Figure 3 below. Table S2 in the Supporting Information, section 5, provides all performance indicators.

**Figure 3:**
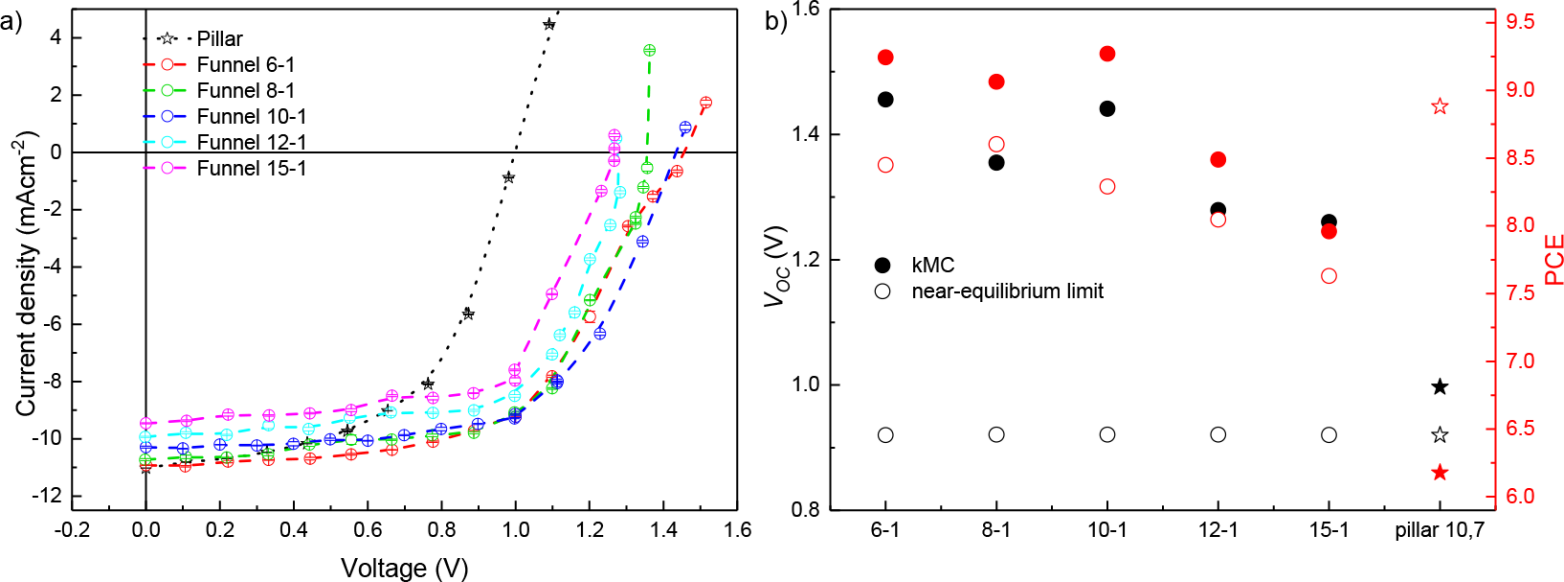
(a) *j*–*V* curves for funnel morphologies with different unit cell sizes and constant tip size. The error bars are of the same size as the symbols. (b) Corresponding *V*_*OC*_ (black) and power conversion efficiency (red) obtained from kMC (solid symbols) and the near-equilibrium limit according to eqs 1–4 (open symbols).

The current–voltage characteristics for different base and unit cell size *m* in Figure 3a show very minor differences at smaller *m*, which we attribute to counteracting changes in efficiency of exciton quenching and subsequent funnel-directed long-range transport to the contacts. Beyond *m* = 10, a more pronounced decay in performance is visible, where both charge generation, reflected in a decreasing *j*_*SC*_, and directed charge motion, reflected in a decreasing *V*_*OC*_, become impeded. We attribute the former to the larger mean distance between D:A interfaces that cause a larger fraction of excitons to recombine; see also the Supporting Information, Figure S8.

The decreasing *V*_*OC*_ at larger *m* may be expected when the typical feature size is no longer much smaller than the typical diffusion distance of the photogenerated charges—in order for the photogenerated charges to “feel” the funnel shape, the diffusion distance within their lifetime has to be much larger than the lateral unit cell size. A very rough estimate of this diffusion distance, given in Supporting Information section 6, gives 40 nm, which is consistent with the observed reduction in *V*_*OC*_ when the unit cell size grows from 6 × 1.8 = 10.8 nm to 15 × 1.8 = 27 nm.

In conclusion, we have shown by numerical calculations that the highly diffusive motion of thermalizing photogenerated charge carriers, which is typical for organic solar cells, can be rectified by funnel-shaped morphologies in the phase-separated donor–acceptor blend. For optimized funnel geometries, the resulting device can break the thermodynamic upper limit for near-equilibrium solar cells. This is possible because the slow thermalization of photogenerated charges does not complete in the charge carrier lifetime, causing the system to be far from equilibrium.

While the current simulations are performed for highly simplified morphologies that will be extremely hard, if not impossible, to realize experimentally using a top-down strategy, we do think the presented results are of significant practical relevance. The reason is that the conditions for the discussed effects to occur, the system being far from equilibrium at all relevant time scales and the funnel feature size being smaller than the diffusion distance, will also be fulfilled for the more convoluted morphologies that are routinely made in real devices. Specifically, we speculate that the spontaneous phase separation occurring in stratified D:A films, as, e.g., produced by sequential deposition of donor and acceptor compounds, will lead to funnel-like networks that resemble those considered here.^33,44,45^ However, for these bottom-up funnels to be maximally effective, both the degree of stratification and the length scale of lateral phase separation will have to be optimized.

## Supporting Information Available

Additional calculations and derivations for homogeneous gradients; details and justification of the kinetic Monte Carlo model; and further numerical results, visualization and discussion (PDF)

## References

[ref1] Cui Y.; Xu Y.; Yao H.; Bi P.; Hong L.; Zhang J.; Zu Y.; Zhang T.; Qin J.; Ren J.; Chen Z.; He C.; Hao X.; Wei Z.; Hou J. Single-Junction Organic Photovoltaic Cell with 19% Efficiency. Adv. Mater. 2021, 33 (41), 2102420 10.1002/adma.202102420. 34464466

[ref2] Chong K.; Xu X.; Meng H.; Xue J.; Yu L.; Ma W.; Peng Q. Realizing 19.05% Efficiency Polymer Solar Cells by Progressively Improving Charge Extraction and Suppressing Charge Recombination. Adv. Mater. 2022, 34 (13), 2109516 10.1002/adma.202109516. 35080061

[ref3] Puttisong Y.; Xia Y.; Chen X.; Gao F.; Buyanova I. A.; Inganäs O.; Chen W. M. Charge Generation via Relaxed Charge-Transfer States in Organic Photovoltaics by an Energy-Disorder-Driven Entropy Gain. J. Phys. Chem. C 2018, 122 (24), 12640–12646. 10.1021/acs.jpcc.8b03432.

[ref4] Wang J.; Yao H.; Xu Y.; Ma L.; Hou J. Recent Progress in Reducing Voltage Loss in Organic Photovoltaic Cells. Mater. Chem. Front. 2021, 5 (2), 709–722. 10.1039/D0QM00581A.

[ref5] van Eersel H.; Janssen R. A. J.; Kemerink M. Mechanism for Efficient Photoinduced Charge Separation at Disordered Organic Heterointerfaces. Adv. Funct. Mater. 2012, 22 (13), 2700–2708. 10.1002/adfm.201200249.

[ref6] Benduhn J.; Tvingstedt K.; Piersimoni F.; Ullbrich S.; Fan Y.; Tropiano M.; McGarry K. A.; Zeika O.; Riede M. K.; Douglas C. J.; Barlow S.; Marder S. R.; Neher D.; Spoltore D.; Vandewal K. Intrinsic Non-Radiative Voltage Losses in Fullerene-Based Organic Solar Cells. Nature Energy 2017, 2 (6), 17053 10.1038/nenergy.2017.53.

[ref7] Shockley W.; Queisser H. J. Detailed Balance Limit of Efficiency of P-n Junction Solar Cells. J. Appl. Phys. 1961, 32 (3), 510–519. 10.1063/1.1736034.

[ref8] Azzouzi M.; Kirchartz T.; Nelson J. Factors Controlling Open-Circuit Voltage Losses in Organic Solar Cells. Trends in Chemistry 2019, 1 (1), 49–62. 10.1016/j.trechm.2019.01.010.

[ref9] Rosenthal K. D.; Hughes M. P.; Luginbuhl B. R.; Ran N. A.; Karki A.; Ko S.-J.; Hu H.; Wang M.; Ade H.; Nguyen T.-Q. Quantifying and Understanding Voltage Losses Due to Nonradiative Recombination in Bulk Heterojunction Organic Solar Cells with Low Energetic Offsets. Adv. Energy Mater. 2019, 9 (27), 1901077 10.1002/aenm.201901077.

[ref10] Felekidis N.; Melianas A.; Kemerink M. The Role of Delocalization and Excess Energy in the Quantum Efficiency of Organic Solar Cells and the Validity of Optical Reciprocity Relations. J. Phys. Chem. Lett. 2020, 11 (9), 3563–3570. 10.1021/acs.jpclett.0c00945. 32301322

[ref11] Melianas A.; Felekidis N.; Puttisong Y.; Meskers S. C. J.; Inganäs O.; Chen W. M.; Kemerink M. Nonequilibrium Site Distribution Governs Charge-Transfer Electroluminescence at Disordered Organic Heterointerfaces. Proc. Natl. Acad. Sci. U.S.A. 2019, 116 (47), 23416–23425. 10.1073/pnas.1908776116. 31690666 PMC6876215

[ref12] Menke S. M.; Ran N. A.; Bazan G. C.; Friend R. H. Understanding Energy Loss in Organic Solar Cells: Toward a New Efficiency Regime. Joule 2018, 2 (1), 25–35. 10.1016/j.joule.2017.09.020.

[ref13] Burke T. M.; Sweetnam S.; Vandewal K.; McGehee M. D. Beyond Langevin Recombination: How Equilibrium Between Free Carriers and Charge Transfer States Determines the Open-Circuit Voltage of Organic Solar Cells. Adv. Energy Mater. 2015, 5 (11), 1500123 10.1002/aenm.201500123.

[ref14] Blakesley J. C.; Neher D. Relationship between Energetic Disorder and Open-Circuit Voltage in Bulk Heterojunction Organic Solar Cells. Phys. Rev. B 2011, 84 (7), 075210–075210. 10.1103/PhysRevB.84.075210.

[ref15] Vandewal K.; Tvingstedt K.; Gadisa A.; Inganäs O.; Manca J. V. Relating the Open-Circuit Voltage to Interface Molecular Properties of Donor:Acceptor Bulk Heterojunction Solar Cells. Phys. Rev. B 2010, 81 (12), 125204 10.1103/PhysRevB.81.125204.

[ref16] Abramavicius V.; Pranculis V.; Melianas A.; Inganäs O.; Gulbinas V.; Abramavicius D. Role of Coherence and Delocalization in Photo-Induced Electron Transfer at Organic Interfaces. Sci. Rep 2016, 6 (1), 32914 10.1038/srep32914. 27605035 PMC5015064

[ref17] Lane P. A.; Cunningham P. D.; Melinger J. S.; Esenturk O.; Heilweil E. J. Hot Photocarrier Dynamics in Organic Solar Cells. Nat. Commun. 2015, 6 (1), 7558 10.1038/ncomms8558. 26179323

[ref18] Bässler H. Charge Transport in Disordered Organic Photoconductors a Monte Carlo Simulation Study. physica status solidi (b) 1993, 175 (1), 15–56. 10.1002/pssb.2221750102.

[ref19] Upreti T.; Wilken S.; Zhang H.; Kemerink M. Slow Relaxation of Photogenerated Charge Carriers Boosts Open-Circuit Voltage of Organic Solar Cells. J. Phys. Chem. Lett. 2021, 12 (40), 9874–9881. 10.1021/acs.jpclett.1c02235. 34609870 PMC8521526

[ref20] Melianas A.; Pranculis V.; Xia Y.; Felekidis N.; Inganäs O.; Gulbinas V.; Kemerink M. Photogenerated Carrier Mobility Significantly Exceeds Injected Carrier Mobility in Organic Solar Cells. Adv. Energy Mater. 2017, 7 (9), 1602143 10.1002/aenm.201602143.

[ref21] Melianas A.; Etzold F.; Savenije T. J.; Laquai F.; Inganäs O.; Kemerink M. Photo-Generated Carriers Lose Energy during Extraction from Polymer-Fullerene Solar Cells. Nat. Commun. 2015, 6 (1), 8778 10.1038/ncomms9778. 26537357 PMC4659933

[ref22] Andersson O.; Kemerink M. Enhancing Open-Circuit Voltage in Gradient Organic Solar Cells by Rectifying Thermalization Losses. Solar RRL 2020, 4 (12), 2000400 10.1002/solr.202000400.

[ref23] Tress W.; Leo K.; Riede M. Effect of Concentration Gradients in ZnPc:C60 Bulk Heterojunction Organic Solar Cells. Sol. Energy Mater. Sol. Cells 2011, 95 (11), 2981–2986. 10.1016/j.solmat.2011.06.003.

[ref24] Beyer B.; Pfeifer R.; Zettler J. K.; Hild O. R.; Leo K. Graded Absorption Layers in Bulk Heterojunction Organic Solar Cells. J. Phys. Chem. C 2013, 117 (19), 9537–9542. 10.1021/jp3109732.

[ref25] Chen L.; Tang Y.; Fan X.; Zhang C.; Chu Z.; Wang D.; Zou D. Improvement of the Efficiency of CuPc/C60-Based Photovoltaic Cells Using a Multistepped Structure. Org. Electron. 2009, 10 (4), 724–728. 10.1016/j.orgel.2009.02.024.

[ref26] Guo X.; Zhou N.; Lou S. J.; Smith J.; Tice D. B.; Hennek J. W.; Ortiz R. P.; Navarrete J. T. L.; Li S.; Strzalka J.; Chen L. X.; Chang R. P. H.; Facchetti A.; Marks T. J. Polymer Solar Cells with Enhanced Fill Factors. Nature Photon 2013, 7 (10), 825–833. 10.1038/nphoton.2013.207.

[ref27] Wang Y.; Wu B.; Wu Z.; Lan Z.; Li Y.; Zhang M.; Zhu F. Origin of Efficient Inverted Nonfullerene Organic Solar Cells: Enhancement of Charge Extraction and Suppression of Bimolecular Recombination Enabled by Augmented Internal Electric Field. J. Phys. Chem. Lett. 2017, 8 (21), 5264–5271. 10.1021/acs.jpclett.7b02308. 29027803

[ref28] Zhang L.; Xing X.; Zheng L.; Chen Z.; Xiao L.; Qu B.; Gong Q. Vertical Phase Separation in Bulk Heterojunction Solar Cells Formed by in Situ Polymerization of Fulleride. Sci. Rep 2015, 4 (1), 5071 10.1038/srep05071. PMC4034005 24861168

[ref29] Huang J.; Carpenter J. H.; Li C.-Z.; Yu J.-S.; Ade H.; Jen A. K.-Y. Highly Efficient Organic Solar Cells with Improved Vertical Donor–Acceptor Compositional Gradient Via an Inverted Off-Center Spinning Method. Adv. Mater. 2016, 28 (5), 967–974. 10.1002/adma.201504014. 26628195

[ref30] Bi P.; Xiao T.; Yang X.; Niu M.; Wen Z.; Zhang K.; Qin W.; So S. K.; Lu G.; Hao X.; Liu H. Regulating the Vertical Phase Distribution by Fullerene-Derivative in High Performance Ternary Organic Solar Cells. Nano Energy 2018, 46, 81–90. 10.1016/j.nanoen.2018.01.040.

[ref31] Pandey R.; Holmes R. J. Graded Donor-Acceptor Heterojunctions for Efficient Organic Photovoltaic Cells. Adv. Mater. 2010, 22 (46), 5301–5305. 10.1002/adma.201002454. 20872410

[ref32] Zhang Y.; Liu K.; Huang J.; Xia X.; Cao J.; Zhao G.; Fong P. W. K.; Zhu Y.; Yan F.; Yang Y.; Lu X.; Li G. Graded Bulk-Heterojunction Enables 17% Binary Organic Solar Cells via Nonhalogenated Open Air Coating. Nat. Commun. 2021, 12 (1), 4815–4815. 10.1038/s41467-021-25148-8. 34376697 PMC8355148

[ref33] Wang H.-C.; Cheng P.; Tan S.; Chen C.-H.; Chang B.; Tsao C.-S.; Chen L.-Y.; Hsieh C.-A.; Lin Y.-C.; Cheng H.-W.; Yang Y.; Wei K.-H. Sequential Deposition of Donor and Acceptor Provides High-Performance Semitransparent Organic Photovoltaics Having a Pseudo p–i–n Active Layer Structure. Adv. Energy Mater. 2021, 11 (13), 2003576 10.1002/aenm.202003576.

[ref34] Weng K.; Ye L.; Zhu L.; Xu J.; Zhou J.; Feng X.; Lu G.; Tan S.; Liu F.; Sun Y. Optimized Active Layer Morphology toward Efficient and Polymer Batch Insensitive Organic Solar Cells. Nat. Commun. 2020, 11 (1), 2855 10.1038/s41467-020-16621-x. 32503994 PMC7275072

[ref35] Li M.; Wang Q.; Liu J.; Geng Y.; Ye L. Sequential Deposition Enables High-Performance Nonfullerene Organic Solar Cells. Materials Chemistry Frontiers 2021, 5 (13), 4851–4873. 10.1039/D1QM00407G.

[ref36] Qin J.; Yang Q.; Oh J.; Chen S.; Odunmbaku G. O.; Ouedraogo N. A. N.; Yang C.; Sun K.; Lu S. Volatile Solid Additive-Assisted Sequential Deposition Enables 18.42% Efficiency in Organic Solar Cells. Advanced Science 2022, 9 (9), 2105347–2105347. 10.1002/advs.202105347. PMC8948555 35072347

[ref37] Nam Y. M.; Huh J.; Jo W. H. Effect of the Vertical Composition Gradient of Active Layer on the Performance of Bulk-Heterojunction Organic Photovoltaic Cell. J. Appl. Phys. 2011, 110 (11), 114521 10.1063/1.3666061.

[ref38] Bi S.; Ouyang Z.; Shaik S.; Li D. Effect of Donor-Acceptor Vertical Composition Profile on Performance of Organic Bulk Heterojunction Solar Cells. Sci. Rep. 2018, 8 (1), 9574–9574. 10.1038/s41598-018-27868-2. 29934618 PMC6014987

[ref39] Abdul Aziz M. Z.; Higashimine K.; Shioya N.; Shimoaka T.; Hasegawa T.; Sakai H.; Vohra V.; Murata H. Controlling the Concentration Gradient in Sequentially Deposited Bilayer Organic Solar Cells via Rubbing and Annealing. RSC Adv. 2020, 10 (61), 37529–37537. 10.1039/D0RA05991A. 35521271 PMC9057144

[ref40] Wilken S.; Upreti T.; Melianas A.; Dahlström S.; Persson G.; Olsson E.; Österbacka R.; Kemerink M. Experimentally Calibrated Kinetic Monte Carlo Model Reproduces Organic Solar Cell Current–Voltage Curve. Solar RRL 2020, 4 (6), 2000029 10.1002/solr.202000029.

[ref41] Felekidis N.; Melianas A.; Kemerink M. Automated Open-Source Software for Charge Transport Analysis in Single-Carrier Organic Semiconductor Diodes. Org. Electron. 2018, 61, 318 10.1016/j.orgel.2018.06.010.

[ref42] Rau U. Reciprocity Relation between Photovoltaic Quantum Efficiency and Electroluminescent Emission of Solar Cells. Phys. Rev. B 2007, 76, 085303–085303. 10.1103/PhysRevB.76.085303.

[ref43] Kirchartz T.; Nelson J.; Rau U. Reciprocity between Charge Injection and Extraction and Its Influence on the Interpretation of Electroluminescence Spectra in Organic Solar Cells. Phys. Rev. Applied 2016, 5 (5), 054003 10.1103/PhysRevApplied.5.054003.

[ref44] Qin J.; Yang Q.; Oh J.; Chen S.; Odunmbaku G. O.; Ouedraogo N. A. N.; Yang C.; Sun K.; Lu S. Volatile Solid Additive-Assisted Sequential Deposition Enables 18.42% Efficiency in Organic Solar Cells. Advanced Science 2022, 9 (9), 2105347 10.1002/advs.202105347. PMC8948555 35072347

[ref45] Li M.; Wang Q.; Liu J.; Geng Y.; Ye L. Sequential Deposition Enables High-Performance Nonfullerene Organic Solar Cells. Mater. Chem. Front. 2021, 5 (13), 4851–4873. 10.1039/D1QM00407G.

