# Supplementary material for: Can Organic Solar Cells Beat the Near-Equilibrium Thermodynamic Limit?
Source: J Phys Chem Lett. 2022 Jul 13;13(28):6514–9. doi: 10.1021/acs.jpclett.2c01565 (PMC9310094; doi:10.1021/acs.jpclett.2c01565)
Supplement: Supplementary file 1 — jz2c01565_si_001.pdf [file jz2c01565_si_001.pdf]

Supplementary Information to

Can Organic Solar Cells Beat the Near-Equilibrium Thermodynamic Limit?

Tanvi Upreti[1,2], Constantin Tormann [2], Martijn Kemerink[1,2]

[1] Complex Materials and Devices, Department of Physics, Chemistry and Biology (IFM), Linköping University, 581 83 Linköping, Sweden

[2] Centre for Advanced Materials, Heidelberg University, Im Neuenheimer Feld 225, 69120 Heidelberg, Germany

E-mail: martijn.kemerink@cam.uni-heidelberg.de

Contents

1 – Open circuit voltage due to homogeneous composition gradients 2

2 – Comparison of homogeneous composition profiles 4

3 – Parameters and detailed balance in the kinetic Monte Carlo model 7

4 – Visualization of funnel morphology 11

5 – Performance characteristics and near-equilibrium upper limit 12

6 – Transient energetics of photocreated charges and calculation of diffusion length 15

7 – Supplementary References 16

1 – Open circuit voltage due to homogeneous composition gradients

In absence of a build-in voltage, the broken inversion symmetry due to the composition gradient still leads to a finite open circuit voltage, which, for not too strong gradients, was found to obey

$$V_{OC} = 2\frac{k_B T_{eff}}{q}\ln\left(\frac{c_{D_0}}{1-c_{D_0}}\right), \quad \text{(S1)}$$

where c_{D_0} and $1 - c_{D_0}$ are the relative fractions of donor material at the anode and cathode, respectively, and T_{eff} is the effective temperature of the charge carrier population.[1]

Equation (S1) can be derived from the Boltzmann expression for the charge carrier density p in the narrow band approximation, which holds for not too large charge carrier densities, viz.

$$p = N_p \exp\left(\frac{q(\varphi_p - V)}{k_B T}\right), \quad \text{(S2)}$$

where $N_p = N_0 c_D(z)$ is the local total density of states and $\varphi_p - V$ is the relative position of the Fermi energy w.r.t. the band energy. Under the assumption that the generation rate of free charges is roughly constant throughout the device and that under open circuit conditions there is no significant charge extraction at the electrodes, the charge carrier density p (in m^{-3}) becomes constant throughout the device at V_{OC}. Specifically, at the extreme ends of the device (contacts), where the concentrations are c_{D_0} and $(1 - c_{D_0})$ respectively, it must hold that

$$p = c_{D_0} N_0 \exp\left(\frac{q(\varphi_{p,1} - V)}{k_B T}\right) = \left(1 - c_{D_0}\right) N_0 \exp\left(\frac{q(\varphi_{p,2} - V)}{k_B T}\right). \quad \text{(S3)}$$

For the difference in chemical potential, it then follows:

$$\frac{c_{D_0}}{1-c_{D_0}} = \exp\left(\frac{q(\varphi_{p,2} - \varphi_{p,1})}{k_B T}\right) \quad \text{(S4)}$$

$$\Delta\varphi_p = \varphi_{p,2} - \varphi_{p,1} = \frac{k_B T}{q}\ln\left(\frac{c_{D_0}}{1-c_{D_0}}\right) \quad \text{(S5)}$$

Taking the effective temperature T_{eff} of the charge carrier population for T, this is the same as Eq. S1 except for a factor 2 that stems from the fact that the same argument holds for the charge carrier of the other polarity.

Equivalently, the same equation can be derived from the Boltzmann transport equation in the relaxation time approximation, which for holes reads [2]

$$j_p = -q\mu_p p \nabla\varphi_p. \quad \text{(S6)}$$

Inserting again the Boltzmann expression for p gives

$$j_p = -qD_p \nabla p + q\mu_p p F_p - qD_p^T p \nabla T_p. \quad \text{(S7)}$$

In absence of temperature and density gradients, the first and last terms on the right-hand side become zero. At V_{OC} one then finds that the effective electric field,

$$F_p = F + \frac{k_B T}{q}\nabla\left(\ln\left(N_p\right)\right) = 0 \quad \text{(S8)}$$

must be zero, where $F = -\nabla V$ is the electric field. Integrating from one contact (with total DOS $c_{D_0} N_0$) to the other (with total DOS $\left(1 - c_{D_0}\right) N_0$) leads to the same expression as above.

In this context, the second part of the right-hand side of Eq. S7 can be interpreted as the local, entropic driving force for charge motion. Integrating in the direction of the composition gradient then gives the entropic potential, viz.

$$V_{entr,p}(z) = \frac{k_B T}{q} \ln\left(\frac{c_{D_0}}{c_D(z)}\right). \qquad \text{(S9)}$$

2 – Comparison of homogeneous composition profiles

In absence of phase separation, the gradient strength is denoted as $100 \times \left(c_{D_0}: 1 - c_{D_0}\right)$, so a 90:10 gradient runs from 90% donor material at the anode to 10% at the cathode, and vice versa for the acceptor material.

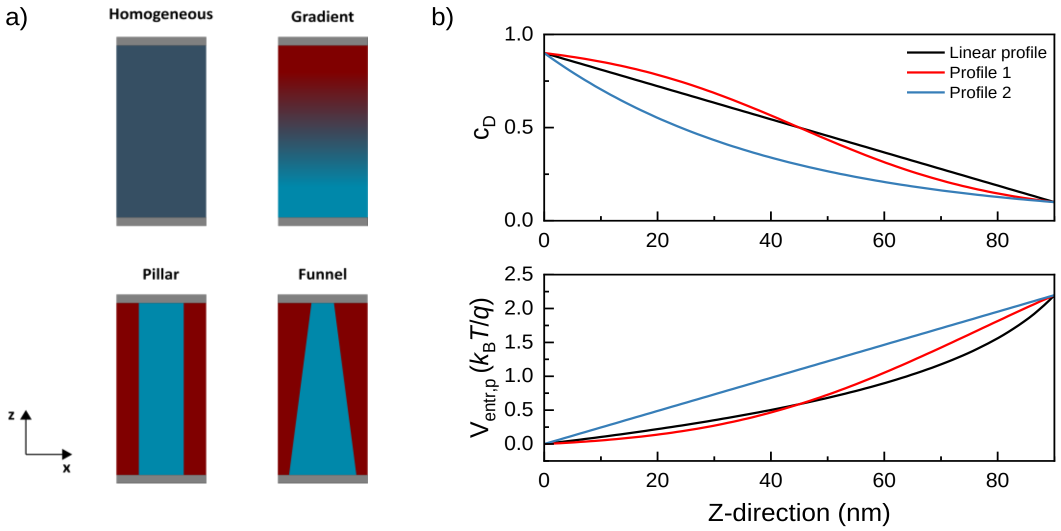

Figure S1: a) Schematics of the different composition profiles considered in this paper. The schematic of the funnel only represents a 2D visualization and not a cross-section, see SI section 4 below for additional visualizations of the funnel morphology. Red and blue regions indicate donor and acceptor material, respectively, the grey regions denote contacts. Dimensions are discussed at the specific calculations. b) Fraction of donor sites along different 90:10 gradient profiles in a 90 nm thick device and their entropic potentials on holes. The mirrored profiles for acceptor sites and entropic potential for electrons are not shown for clarity.

Profile shapes

Apart from the linear profiles discussed by Andersson[1], we also considered two nonlinear gradient profiles, the first of which has the shape of the Fermi distribution as a proxy of a profile with typical width W (here taken to be 18 nm, Figure S1) that could result from annealing an abrupt bilayer halfway the device of thickness L,[3]

$$c_D(z) = \frac{1}{2} - \frac{2c_{D0}}{c_{Norm}}\left(\frac{1}{\exp((z-L/2)/W)+1} - \frac{1}{2}\right), \quad \text{(S10)}$$

where L is the device thickness and c_{Norm} is a normalization factor given by

$$c_{Norm} = 1 - 2/\left(1 + \exp\left(\frac{L}{2W}\right)\right) \quad \text{(S11)}$$

The drawback of both the linear profile and the curved profile 1 from Eq. (S10) is that the driving force for charge motion drops significantly towards the extracting electrode. The latter can be seen in Figure S1b where we plotted the corresponding entropic potential, $V_{entr,p} = (k_B T/q)\log\left(c_{D_0}/c_D(z)\right)$, which reflects the position-dependence of the available site density for holes, c.f. SI Eq. S9.

Having a constant gradient in V_{entr} for both electrons and holes, see profile 2 in Figure S1b, is impossible in a two-component DA-blend. This follows from the shape of the (donor) profile 2 in panel b that can be calculated by inverting the above expression for $V_{entr,p}$, assuming the shown linear potential profile: in a two-component

blend, the corresponding acceptor profile $c_A(z) = 1 - c_D(z)$ has another form than the donor profile and thus does not give a linear potential profile $V_{entr,n}$. Hence, having constant gradients for both electrons and holes requires a third, otherwise inactive compound – although this might be an absorbing compound that transfers charge or energy to the primary donor and/or acceptor, its sites must be inaccessible to the mobile charges to not perturb the linear gradients in V_{entr}. Although such a ternary blend might, in principle, be fabricated by co-evaporation of suitably chosen compounds, we consider this 3-component profile as instructive but hypothetical. The functional form obtained by inverting the linear entropic potentials, defining profile 2, is

$$c_2(z) = \exp\left(\frac{\ln c_{D_0} + \ln(1 - c_{D_0})}{2} + \left(z - \frac{L}{2}\right)\frac{\ln(1 - c_{D_0}) - \ln c_{D_0}}{L}\right) \qquad (S12)$$

Results

Figure S2a compares the calculated current-voltage (jV) characteristics for devices with different concentration gradient profiles and $V_{bi} = 0$. In absence of a built-in voltage, the nonzero V_{OC} and j_{SC} values are entirely due to the composition gradient, also leading to jV-curves that show a typical linear voltage dependence that was previously reported in Ref. [4]. Irrespective of the profile shape, the open circuit voltage increases with increasing gradient strength, and follows the dependence predicted by Eq. S1, see Figure 2b. The deviations at higher concentrations can be attributed to carrier blocking effects that are not accounted for in S1.[4] The differences between the linear and Fermi profile Eq. S10 can be understood in terms of a faster extraction in case of the latter profile, which leads to a reduced (time for) thermalization and a somewhat higher effective temperature T_{eff}.

Although it offers the highest V_{OC} values, the 3-component profile 2, Eq. S12, leads to significantly reduced short circuit current densities, which can be attributed to an enhanced exciton recombination, see Figure S3 in the SI, which we attribute to the relatively wide regions with unbalanced donor:acceptor compositions in combination with the presence of a third compound that blocks final sites for charge transfer.

As may be expected, the performance of devices based on the Fermi profile 1 depends on the width W, see Figure S4.

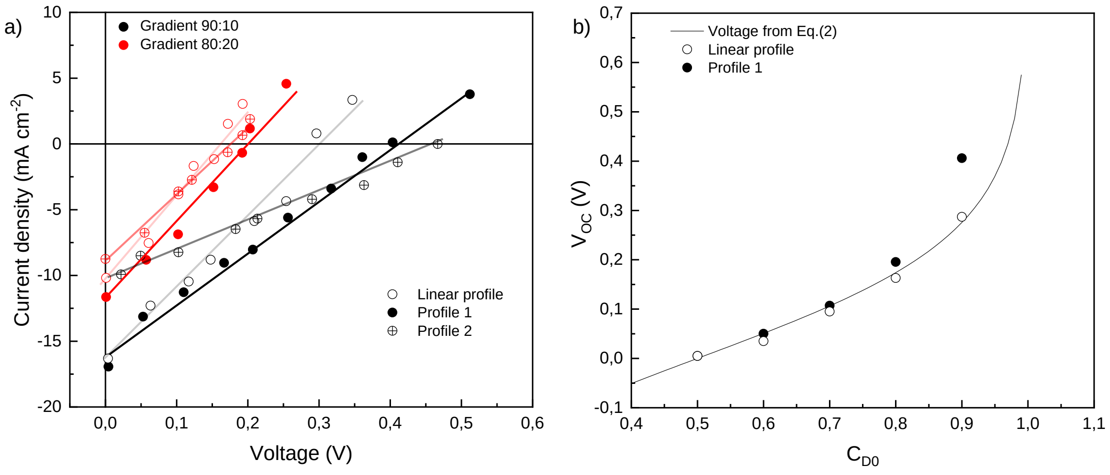

Figure S2: a) Comparison of jV-curves for linear and curved composition profiles for an 80:20 (black) and 90:10 (red) gradient. b) Open circuit voltage vs. gradient strength. Symbols are kMC calculations, lines are guides to the eye (panel a) resp. fits to Eq. (2) with $T_{eff} = 728$ K (panel b). For all calculations, $V_{bi} = 0$.

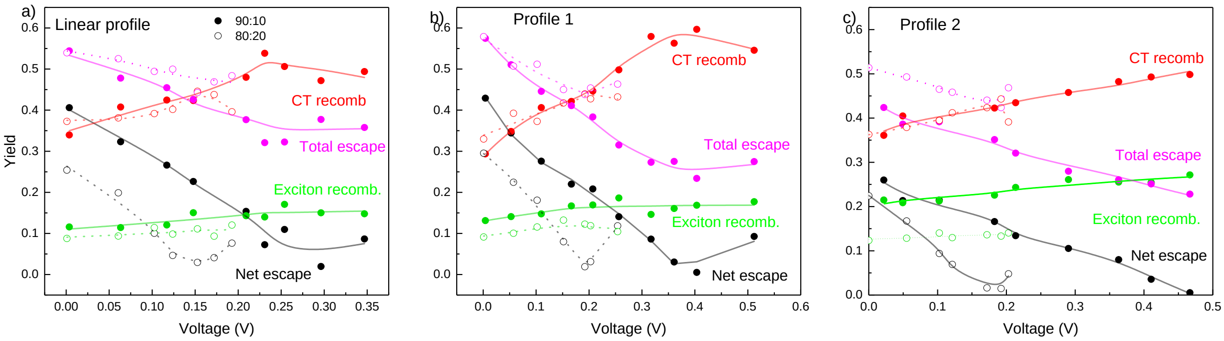

Figure S3: Yields for the same gradient concentration profiles (linear, Profile-1, Profile-2) as in Figure S2. Profile 2 (3-component, constant entropic driving force) shows the strongest exciton recombination and hence the lowest short circuit current. The lines are guides to the eye.

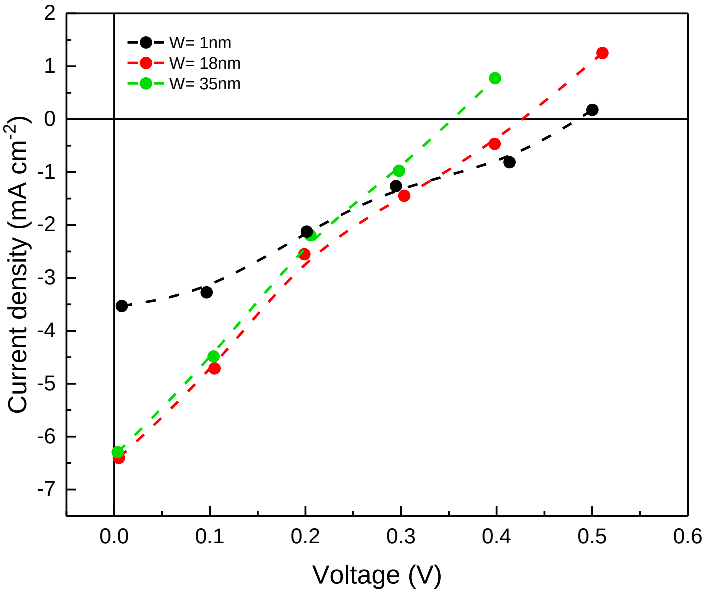

Figure S4: Calculation of the impact of width W, as it appears in Eq. S10 for a 90:10 gradient (Fermi profile 1). For a narrow junction (black symbols), the device effectively becomes a sharp bilayer, showing the characteristic high V_{OC} and low J_{SC} values.

3 – Parameters and detailed balance in the kinetic Monte Carlo model

Table S1: Overview of the parameters used for KMC simulations.

Parameter [unit]	Value[5]
Nearest neighbor distance, a_{NN} [nm]	1.8
$E_{LUMO,acceptor}$ [eV]	3.7
$E_{HOMO,donor}$ [eV]	5.3
Energetic disorder electrons, σ_e [meV]	75
Energetic disorder holes, σ_h [meV]	75
Attempt-to-hop frequency electrons, $\nu_{0,e}$ [s-	1e11
Attempt-to-hop frequency holes, $\nu_{0,h}$ [s^{-1}]	1e11
CT recombination rate, ν_{CT} [s^{-1}]	3e7
Exciton recombination rate, ν_{S1} [s^{-1}]	1e9
Injection barrier height [eV]	0.3

In the following, we shall discuss how the used kinetic Monte Carlo model is consistent with the concept of detailed balance and why it can be meaningfully compared to the near-equilibrium model outlined in the main text (Eqs. 1-4).

The principle of detailed balance between photogeneration and radiative recombination prescribes that the following relation is obeyed in equilibrium:[6]

$$\int \phi_{abs}(E)\phi_{\mathrm{BB}}(E)dE = k_{rad}n_i^2 \qquad \text{(S13)}$$

where k_{rad} is the bimolecular radiative recombination rate and n_i the intrinsic charge carrier density. For the kMC model used, a problem arises since the bimolecular recombination constant k_{rad} is not an explicit parameter; instead, it is emergent and even in equilibrium it depends on the hopping parameters, morphology and disorder.[7] In absence of an analytical expression for k_{rad}, this means one would have to determine k_{rad} for each simulated system by cumbersome 'reverse engineering'. A more fundamental problem is that in organic systems, bimolecular recombination is typically understood in terms of an (encounter-limited) Langevin model, which would lead to the unphysical situation that mobilities would have to depend on the absorption spectrum if Eq. S13 to hold. We are not aware of a formal solution to this problem but assume it to be related to the fact that at typical values of n_i (~4e14 m^{-3} for the HOMO and LUMO bands in the system discussed here), the associated inter-particle distance (~10 micron) is much larger than the diffusion distance, making the recombination of thermally excited electrons and holes effectively unimolecular. In other words, the actual environment that a localized charge sees in a kMC simulation, or an actual localized charge in an organic semiconductor, is very different from the mean density n_i that for example enters in drift-diffusion simulations.

Rather than setting a macroscopic bimolecular recombination coefficient, we therefore use a microscopic argument to arrive at a radiative lifetime. In detailed balance, for the equilibrium transition rates between any two states i and j with energy $E_{i,j}$, it must hold that

$$\frac{\nu_{i\to j}}{\nu_{j\to i}} = \exp\left(\frac{E_i - E_j}{k_B T}\right) \qquad \text{(S14)}$$

The used Miller-Abrahams rates, and thereby the full charge transport, including from and to the contacts, fulfill this requirement. To apply this to the balance between thermal generation and radiative recombination, we use the fact that, in the simulations, the absorption by the active layer is proportional to the joint density

of states and the recombination rate. Then, the excitation current density at a given energy, that is the argument of Eq. 2, becomes simply

$$j_0/q = \phi_{abs}(E)\phi_{\mathrm{BB}}(E)dE \tag{S15}$$

Applying this to the S1 absorption maximum, for which $E_{S1} = 1.5$ eV (2.0 eV bandgap minus 0.5 eV exciton binding energy) and $\phi_{abs}(E_{S1}) = 1$ (c.f. Eqs .3 and 4 in the main text), one finds $j_0/q \approx 141\, m^{-2}s^{-1}$. To arrive at the rate for a single absorption site, we divide by the corresponding 2D (joint) density of states,

$$g_{j,2D}(E) = \frac{N_0}{\sqrt{2\pi\sigma_j^2}} \exp\left(-\frac{(E-E_{S1})^2}{2\sigma_j^2}\right) LdE \tag{S16}$$

with $N_0 = a_{NN}^{-3}$ the total site density, L the device thickness (here 90 nm) and $\sigma_j^2 = \sigma_{LUMO}^2 + \sigma_{HOMO}^2$ the width of the joint DOS. Hence, for the S0→S1 absorption, one has

$$\nu_{S0\rightarrow S1} = \frac{\phi_{\mathrm{BB}}(E_{S1})}{g_{j,2D}(E_{S1})} \tag{S17}$$

Using Eq. S14, the detailed balance value for the radiative recombination rate can be calculated to be $k_{rad} = \nu_{S1\rightarrow S0} = 0.04 \times 10^9\, s^{-1}$. This is considerably (~25 times) slower than the inverse exciton lifetime of $\nu_{S1} = 1 \times 10^9\, s^{-1}$ that we employed in the simulations. Note that for both ν_{S1} and ν_{CT} experimentally calibrated values have been used, as further discussed below.

The same calculation as above can be made for all other energies, leading to essentially the same result, i.e. a factor ~25 difference between the radiative recombination rate that follows from reciprocity and the rate used in kMC, due to the proportionality between absorption and the joint DOS and recombination rate (ν_{CT} or ν_{S1}). As such, the V_{OC} values from the kMC simulations are a lower limit; depending on whether the recombination is encounter- or lifetime-limited, the deviations might become more, or less, important.

To test the importance of the radiative lifetime for the calculations shown herein, we repeated a representative jV-curve calculation with both ν_{CT} and ν_{S1} reduced by a factor 25. The result is shown in Figure S5 below. Although the fill factor and short circuit current density are slightly increased, the open circuit voltage remains unaffected within numerical accuracy. Hence, for the used parameters, the device operates in the encounter-limited recombination regime, making it largely insensitive to the recombination rates used.

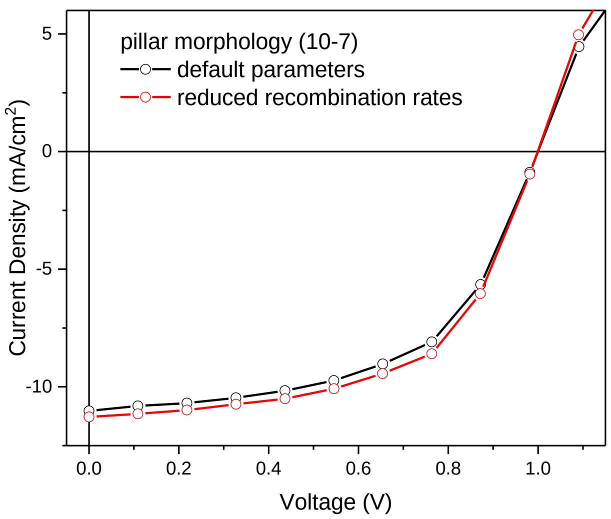

Figure S5: Simulation of the jV-curve of the OPV device with 10-7 pillar morphology and default parameters (black line and points) and the same for ν_{CT} and ν_{S1} reduced by a factor 25 (red line and points). Error bars are of the size of the data points.

A further reason to use the experimentally calibrated values for the recombination rates is that it assures that the calculated results are transferable to realistic materials, provided the right morphology has been realized. Note also that the factor ~25 difference between ν_{S1} and $\nu_{S1\rightarrow S0}$ agrees well with typical EQE values of polymer OLEDs, which are in the few percent range for emitters that show high quantum efficiencies in solution.[8]

Summarizing, the used recombination constants and the methodology used in kMC are consistent with detailed balance in the regime where both radiative and non-radiative processes contribute. The used recombination rates are dominated by the latter, and therefore the V_{OC} values from the kMC simulations are lower limits to what would be formally achievable.

It should be noted that n_i, c.f. Eq. S13, is not explicitly included in the model as a dark-generation factor. The reason is that the associated number of charge carriers is negligible: for the typically used box size, n_i times the box volume is ~1e-7. Accordingly, the thermal generation rate is so low that not a single generation event is to be expected in any doable simulation. This also follows from a comparison of the dark saturation current density $j_0 \approx 7 \times 10^{-14}$ A/m^2 while the device current is around 100 A/m^2. However, n_i is implicitly accounted for since the active layer is coupled to Ohmic contacts that, in equilibrium, inject charge carrier distributions that are consistent with Fermi-statistics. This was explicitly checked.

It has been argued that for a formal comparison of V_{OC} values from device models to the thermodynamic near-equilibrium limit, the former have to account for photon recycling, that is, reabsorption of radiatively emitted photons.[9] In principle, one might, in the kMC model, map the effects hereof on a reduced k_{rad}. In view of the above discussed insensitivity of our results to this parameter, a minor lowering of the radiative lifetime due to photon recycling will not significantly affect our results.

A further test of our model would be to artificially speed up the thermalization. Unfortunately, there is no independent parameter in kMC to do so while keeping everything else the same. Specifically, the system is invariant to increasing the attempt to hop frequency as this also (linearly) scales the mobilities and thereby the extraction rate. Likewise, increasing the layer thickness to increase the extraction time and thereby the degree of thermalization is numerically impossible due to the weak logarithmic dependence of thermalization on (extraction) time. Although this has some drawbacks, we therefore reduced the energetic disorder to a

value $\sigma_{e/h} = 0.001\ \text{eV} \ll k_B T$ such that a band-like model should result.[1] Doing this, while keeping all other parameters unchanged,[2] leads to a V_{OC} value of ~1.21 eV, which is actually slightly below the corresponding near-equilibrium prediction of 1.25 V. We attribute the slightly lower simulated 'band-like' V_{OC} value to the presence of contacts in a device with a rather thin (90 nm) active layer: upon simulating a conventional OPV device in Ref. [10], we found a similar deviation when comparing V_{OC} from a drift-diffusion model (0.66 V) to the value from essentially the same analytical near-equilibrium model as used here (0.69 V).[3]

[1] The problem with this procedure is that, due to the used Miller-Abraham rates, at forward field the charge carrier velocity is no longer connected to the electric field as $v = \mu E$ but instead becomes limited by the rate of downward hops, i.e. $v = a_{NN}\nu_0 = 180\ m^2 s^{-1}$ for the parameters used. This leads to a 'mobility' $\mu = a_{NN}\nu_0 / E$ that is inversely proportional to the electric field, which is unphysical. While this does affect the coupling to the contacts, it should formally not affect the fulfilment of detailed balance.

[2] We set the injection barriers to 0.1 eV (instead of 0.3 eV) to prevent V_{OC} from becoming limited by the built-in voltage.

[3] Running a drift-diffusion simulation with the reduced disorder as input parameter actually leads to much lower Voc than found by the kMC model; only when the mobility is artificially set to a very low value, or the device is made very thick, that is, when the coupling to the contacts is artificially reduced, we do recover the near-equilibrium value.

4 – Visualization of funnel morphology

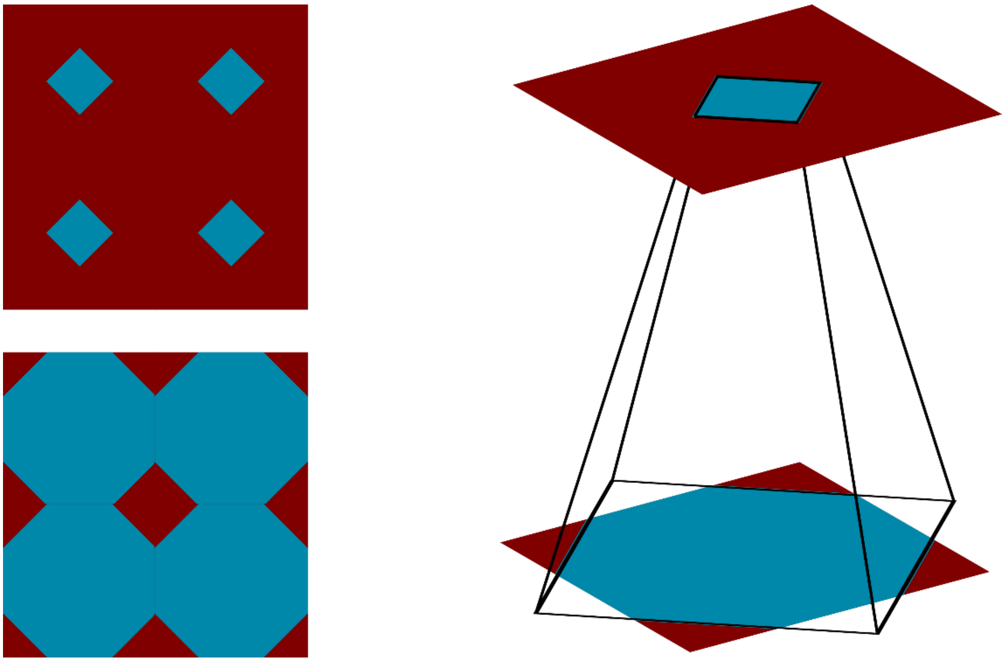

Figure S6: a) Top and bottom view of four adjacent 10x10 unit cells of donor (red) and 3x3 inclusions of acceptor (blue) and b) 3D visualization of the corresponding funnel structure.

5 – Performance characteristics and near-equilibrium upper limit

The parameters extracted from the jV-curves simulated by kMC and from the near-equilibrium calculation (Eqs. 1-4 in the main text) are summarized in table S2 below. The fill factor (FF) is obtained from the jV-curve as calculated from the Shockley equation

$$J = j_0 \left[\exp\left(\frac{qV}{k_B T}\right) - 1 \right] - j_{SC} \tag{S13}$$

The reverse dark saturation current j_0 is calculated as described in the main text.

Table S2: Detailed overview of the performance of solar cell devices as obtained via kMC modelling and as calculated from the approach outlined in the main text (Eqs. 1-4). The latter numbers are marked by an asterix (*). The corresponding jV-curves are shown in Figure S8; see also Figure S9 where the performance indicators are plotted vs. geometry.

Morphology	V_{oc} (V)	J_{sc} (mA/cm2)	FF	η (%)	V_{oc}* (V)	FF*	η* (%)
Funnel 6-1	1.46	108.93	0.58	9.24	0.92	0.84	8.45
Funnel 8-1	1.36	106.68	0.63	9.06	0.92	0.87	8.60
Funnel 10-0	1.43	96.66	0.62	8.54	0.92	0.87	7.77
Funnel 10-1	1.44	102.88	0.63	9.27	0.92	0.87	8.29
Funnel 10-2	1.12	100.80	0.65	7.25	0.92	0.87	8.11
Funnel 10-3	1.09	105.79	0.63	7.31	0.92	0.87	8.52
Funnel 12-1	1.28	99.89	0.66	8.49	0.92	0.87	8.04
Funnel 15-0	1.23	92.59	0.69	7.85	0.92	0.87	7.44
Funnel 15-1	1.26	94.89	0.67	7.96	0.92	0.87	7.63
Funnel 15-2	1.12	95.32	0.66	6.98	0.92	0.87	7.67
Funnel 15-3	1.10	97.63	0.65	6.93	0.92	0.87	7.86
Pillar 10-7	1.00	110.28	0.56	6.18	0.92	0.87	8.88
Pillar 15-7	1.00	98.53	0.56	5.52	0.92	0.87	7.94
Gradient (90:10)	1.35	98.11	0.44	5.82	0.90	0.86	7.60
Bilayer	1.30	61.13	0.44	3.49	0.91	0.87	4.85
No morphology	1.00	99.34	0.41	4.07	0.90	0.87	7.77

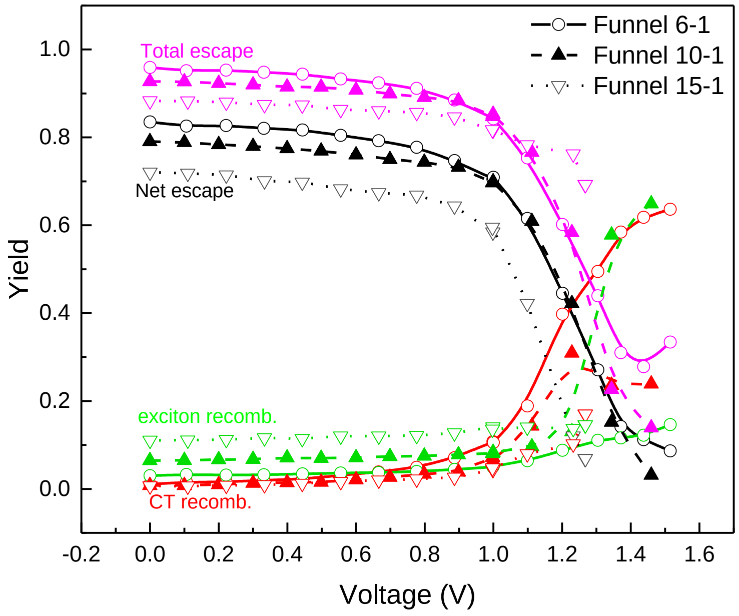

Figure S7: Yields of funnel 6-1(solid lines and open circles), funnel 10-1 (dashed lines and closed up triangles) and funnel 15-1 (dotted lines and open down triangles). Total and net escape yields are defined as $y_{\text{total}} = (J_{n,\text{an}} + J_{n,\text{cat}} + J_{p,\text{an}} + J_{p,\text{cat}})/J_{\text{abs}}$ and $y_{\text{net}} = (-J_{n,\text{an}} + J_{n,\text{cat}} + J_{p,\text{an}} - J_{p,\text{cat}})/J_{\text{abs}}$, where $J_{(n/p),(\text{an/cat})}$ is the current density of photo-generated electrons/holes extracted via the anode/cathode and J_{abs} is the current density corresponding to light absorption. The curves labeled exciton and CT recombination show the relative current densities associated with exciton and CT recombination, i.e., the fraction of photo-generated charges that undergo these processes.

We attribute the fact that the 10-1 funnel is optimal, c.f. Figure 3 in the main text, to the presence of at least two counteracting effects. First, the increased base size, with constant apex size, leads to a stronger funneling action. However, as visible in Figure S7, with increasing funnel base size, exciton recombination becomes an increasingly significant loss mechanism due to the associated increasing distances to an interface where charge transfer can happen.

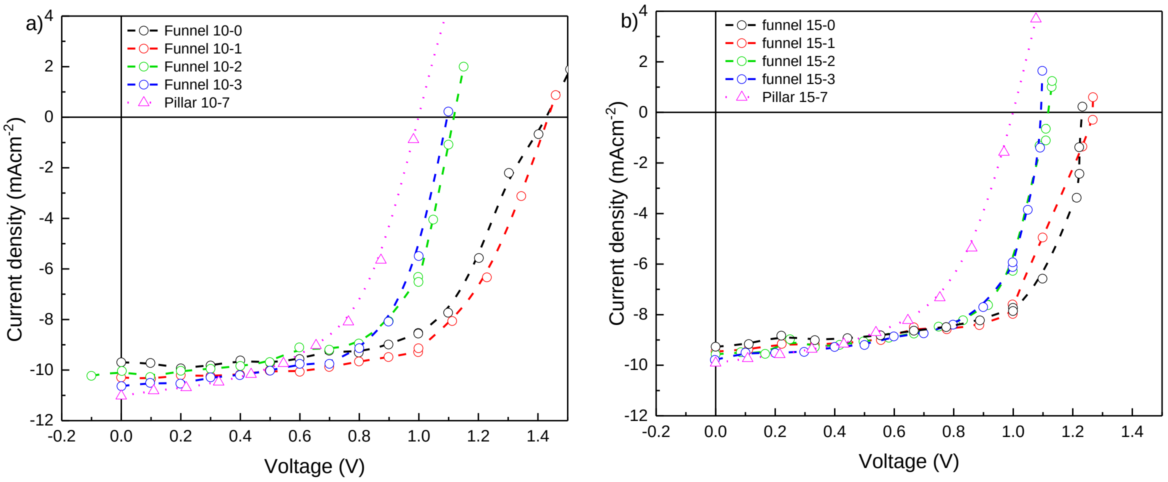

Figure S8: jV curves for funnel morphologies with base size *m* = 10 (a) and *m* = 15 (b) with different apex sizes *n*. For comparison, the pillar morphology is shown as well (open triangles and dotted line). Error bars are of the size of the symbols.

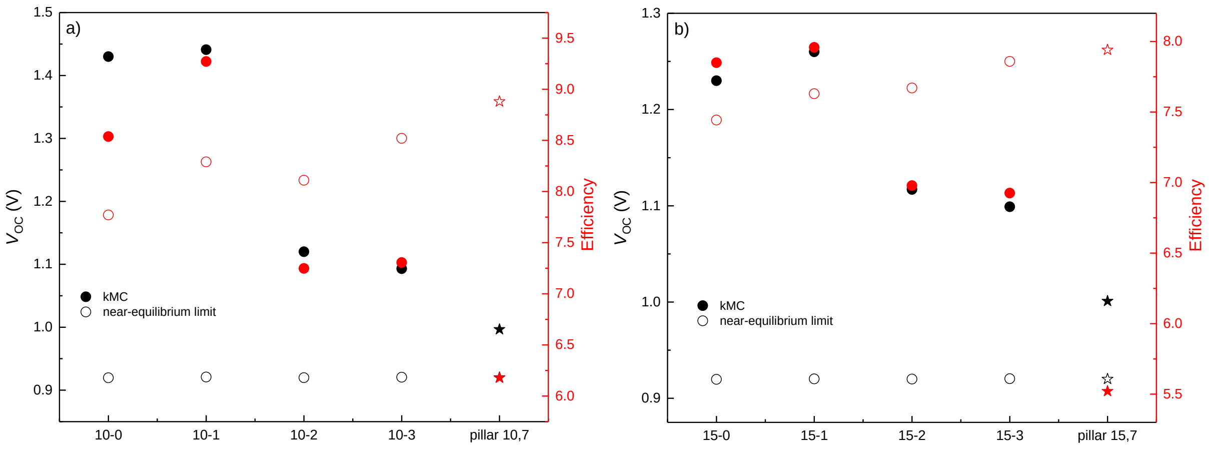

Figure S9: Comparison of V_{OC} and power conversion efficiency of the devices shown in Figure S8 above with their corresponding near-equilibrium limit.

6 – Transient energetics of photocreated charges and calculation of diffusion length

A rough estimate for the typical diffusion length of photocreated charges in the investigated material, in absence of any morphology, can be calculated from

$$L_D = \sqrt{6D\tau} \quad \text{(S14)}$$

where the diffusion constant D is estimated from the mobility using the Einstein relation $D = \mu k_B T/q$. We estimate the typical lifetime τ of the charges from the extraction time histograms; an example of such a plot is shown in Figure S10a, b. Although these plots do not account for charge carrier recombination, it follows from the yield plots in Figure S7, which show that even around open circuit conditions the majority of photocreated charges reaches an electrode [10], that the time at which the normalized cumulative extracted charge reaches ~0.5 is a relevant measure for the lifetime. Both for short and open circuit conditions, this time is around $\tau \approx 10^{-8}$ s. Note that at this time, charge thermalization has not yet completed, as can be seen from the fact that the mean electron and hole energies (solid lines, left y-axis in panel c) have not reached the equilibrium energy (dashed lines). To account for the 'hotness' of the charge carriers at this time, we use the transient charge carrier mobility $\mu(\tau) \approx 10^{-6}$ m^2/Vs as shown by the fine dotted lines in panel c of Figure S10. Note that the kMC parameters in Table S1 lead to a much lower equilibrium mobility of 10^{-7} m^2/Vs.[11] Combined, these numbers lead to $L_D \approx 40$ nm.

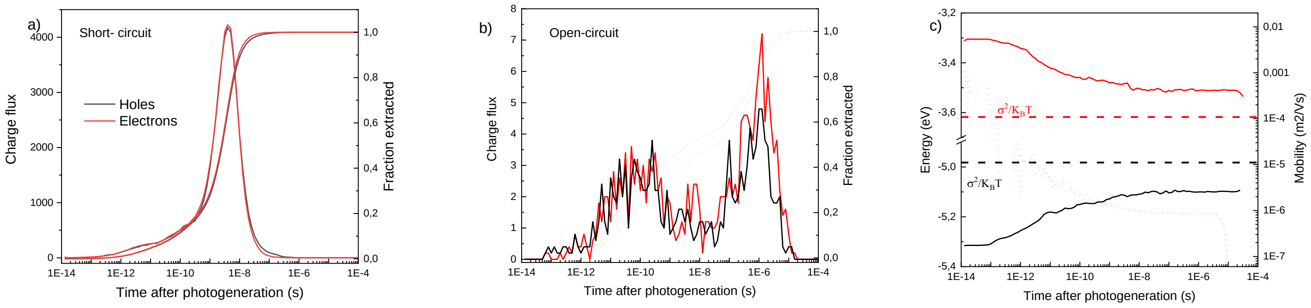

Figure S10: Extraction time distribution of photogenerated electrons (red) and holes (black) as solid lines, the corresponding integrated fraction of extracted charge is shown as dotted lines for funnel morphology (10-1) at short circuit (panel a) and open circuit (panel b) conditions. Panel (c) shows the corresponding thermalization of photogenerated charges, the dashed horizontal lines (red and black) indicate the equilibrium energies that sits $\sigma^2/k_B T$ below (above) the LUMO (HOMO) energy. All calculations for panel a and b are done under steady state conditions and are thus relevant to device operation. The fine dotted lines in panel c show the corresponding transient mobility (right y-axis) in the homogeneous material after a light pulse.

7 – Supplementary References

(1) Andersson, O.; Kemerink, M. Enhancing Open-Circuit Voltage in Gradient Organic Solar Cells by Rectifying Thermalization Losses. *Solar RRL 4* (12), 2000400. https://doi.org/10.1002/solr.202000400.

(2) Selberherr, S. A Glimpse on Results. In *Analysis and Simulation of Semiconductor Devices*; Selberherr, S., Ed.; Springer: Vienna, 1984; pp 258–285. https://doi.org/10.1007/978-3-7091-8752-4_9.

(3) Zawodzki, M.; Resel, R.; Sferrazza, M.; Kettner, O.; Friedel, B. Interfacial Morphology and Effects on Device Performance of Organic Bilayer Heterojunction Solar Cells. *ACS applied materials & interfaces* **2015**, *7* (30), 16161-16168-16161–16168. https://doi.org/10.1021/acsami.5b04972.

(4) Andersson, O.; Kemerink, M. Enhancing Open-Circuit Voltage in Gradient Organic Solar Cells by Rectifying Thermalization Losses. *Solar RRL* **2020**, *4* (12), 2000400. https://doi.org/10.1002/solr.202000400.

(5) Wilken, S.; Upreti, T.; Melianas, A.; Dahlström, S.; Persson, G.; Olsson, E.; Österbacka, R.; Kemerink, M. Experimentally Calibrated Kinetic Monte Carlo Model Reproduces Organic Solar Cell Current–Voltage Curve. *Solar RRL* **2020**, *4* (6), 2000029. https://doi.org/10.1002/solr.202000029.

(6) van Roosbroeck, W.; Shockley, W. Photon-Radiative Recombination of Electrons and Holes in Germanium. *Phys. Rev.* **1954**, *94* (6), 1558–1560. https://doi.org/10.1103/PhysRev.94.1558.

(7) Zuo, G.; Shoaee, S.; Kemerink, M.; Neher, D. General Rules for the Impact of Energetic Disorder and Mobility on Nongeminate Recombination in Phase-Separated Organic Solar Cells. *Phys. Rev. Applied* **2021**, *16* (3), 034027. https://doi.org/10.1103/PhysRevApplied.16.034027.

(8) Burns, S.; MacLeod, J.; Trang Do, T.; Sonar, P.; Yambem, S. D. Effect of Thermal Annealing Super Yellow Emissive Layer on Efficiency of OLEDs. *Sci Rep* **2017**, *7* (1), 40805. https://doi.org/10.1038/srep40805.

(9) Rau, U.; Paetzold, U. W.; Kirchartz, T. Thermodynamics of Light Management in Photovoltaic Devices. *Phys. Rev. B* **2014**, *90* (3), 035211. https://doi.org/10.1103/PhysRevB.90.035211.

(10) Upreti, T.; Wilken, S.; Zhang, H.; Kemerink, M. Slow Relaxation of Photogenerated Charge Carriers Boosts Open-Circuit Voltage of Organic Solar Cells. *J. Phys. Chem. Lett.* **2021**, *12* (40), 9874–9881. https://doi.org/10.1021/acs.jpclett.1c02235.

(11) Pasveer, W. F.; Cottaar, J.; Tanase, C.; Coehoorn, R.; Bobbert, P. A.; Blom, P. W. M.; de Leeuw, D. M.; Michels, M. A. J. Unified Description of Charge-Carrier Mobilities in Disordered Semiconducting Polymers. *Physical Review Letters* **2005**, *94* (20). https://doi.org/10.1103/PhysRevLett.94.206601.
